# Downstream of β-catenin: A positive feedback loop between Goosecoid and Nodals in Spemann’s organizer

**DOI:** 10.1073/pnas.2610127123

**Published:** 2026-09-24

**Authors:** Yagmur Azbazdar, Edward M. De Robertis

**Affiliations:** ^a^ Department of Biological Chemistry, David Geffen School of Medicine, University of California, Los Angeles, CA 90095-1662

**Keywords:** Goosecoid, Nodal, chordin, Siamois, β-catenin

## Abstract

We report on the role of the homeobox gene *goosecoid* (*gsc*) within an epistatic pathway that mediates body axis formation in *Xenopus*. Embryos depleted of β-catenin form exclusively ventral structures and lack all traces of axial structures. Microinjection of *gsc* mRNA rescues an entire body axis with head and trunk-tail. This remarkable activity is shared with the homeobox gene *siamois* (*sia*) and growth factors of the Nodal family. Using a Gsc antisense morpholino, we showed that dorsalization by *Xenopus* nodal-related 6 mRNA has a strong requirement for Gsc in β-catenin depleted embryos. These experiments uncovered a positive feedback loop between Gsc and Nodal that is required downstream of *β-catenin* for the maintenance of dorsal and anterior tissues during gastrulation in endomesoderm. The Gsc-Xnr feedback loop leads to the expression of Spemann organizer genes that antagonize signals from ventral tissues. Similarly, rescue of β-catenin depletion by murine *β-catenin* mRNA also has a requirement for Gsc. Although *gsc* is a gene conserved throughout the animal kingdom during evolution, its loss-of-function during mouse gastrulation has only very weak phenotypic effects; perhaps the redundancy described here in *Xenopus* might be part of the explanation. The functional pathway defined here is self-organizing and robust as required to form a perfect embryo time after time.

## Significance

The vertebrate embryo requires resilient mechanisms to generate a perfect body shape time after time. A homeobox gene called *goosecoid (gsc)* is expressed in the anterior of early embryos throughout the animal kingdom. Here, we report that microinjection of *gsc* mRNA can rescue a complete body axis in embryos that lack one due to the depletion of β-catenin. Other genes such as *siamois* and growth factors known as Nodals share this remarkable activity, indicating a redundant pathway leading to the expression of downstream genes such as *chordin* that mediate the differentiation of central nervous system and dorsal mesoderm. In this study, we identified a positive feedback loop between Goosecoid and Nodals that helps specify Spemann organizer tissue during early development.

The isolation of the Spemann organizer gene *goosecoid* (*gsc*) initiated a new era in the molecular dissection of embryonic induction (1, 2). This gene is expressed in a 60^o^ arc of the dorsal side of the *Xenopus* embryo, the region that exhibits strong inducing activity when transplanted into the ventral side of a host embryo. *gsc* represents the archetypal organizer gene, and the descendants of *gsc*-expressing cells at the gastrula stage give rise to the foregut endomesoderm, prechordal plate, notochord, and chordoneural hinge (CNH) which classically mark the head and trunk-tail organizers (3). In gastrula stage, embryos, dorsal mesoderm plays a crucial inductive role in directing overlying ectodermal cells toward a neural fate (4, 5). Consistent with its expression in dorsal mesoderm, microinjection of *gsc* mRNA induces incomplete secondary body axes through the upregulation of organizer-associated genes such as *chordin* (*chd*) and *noggin* (4).

Goosecoid not only regulates fundamental developmental processes but is also deeply conserved throughout animal evolution, from *Hydra* to humans (6, 7). Therefore, it was a big surprise when knockout of mouse *gsc* resulted in a lack of gastrulation phenotypes, with pups dying at birth but with only small axial reductions in the base of the cranium (8–10). Gsc has a postgastrulation phase of expression in the craniofacial region, which is strongly affected in mice and human Gsc loss-of-function mutations but unrelated to its early phase of expression (10, 11). In *Drosophila*, loss-of-function of *gsc* also lacks gastrulation phenotypes but is an embryonic lethal affecting foregut development (12). In *Xenopus* embryos, antisense RNA or antimorphic *gsc* constructs interfere with head formation (13, 14). In *Xenopus laevis*, which has two copies of *gsc* resulting from allotetraploidy, an antisense morpholino targeting both pseudoalleles causes reductions in head structures that, interestingly, are rescued by knockdown of the ventral homeobox genes *Vent1* and *Vent2* which are targets of BMP4 (15). Why a highly conserved gastrulation gene with copious expression has only weak loss-of-function phenotypes remains an unsolved mystery.

Recently, a cytoplasmic determinant gene called *huluwa* (*hwa*) was discovered by the laboratory of Anming Meng (16). It encodes a transmembrane protein that stabilizes β-catenin on the dorsal side of zebrafish and *Xenopus* embryos by inhibiting Glycogen Synthase Kinase 3 (GSK3) and promoting Axin1 degradation (16, 17). Hwa function requires membrane trafficking and lysosomal activity (18). Microinjected *Xenopus* hwa mRNA induces secondary axes complete with eyes with a penetrance of almost 100% of embryos, unlike other reagents in the β-catenin pathway. In previous studies, we took advantage of *hwa* mRNA microinjections to determine an epistatic molecular pathway for axial patterning by the Spemann organizer (19). Each step of this signaling pathway was established by gain- or loss-of-function experiments using coinjection of mRNAs, secreted antagonists, or antisense morpholino (MO) reagents. Using β-catenin depleted embryos, which provide a sensitized system that also has almost full penetrance (20), we found that Nodals could rescue a complete dorsal axis even at very low doses (19). In addition to Nodals, dorsal β-catenin independently induces Siamois (Sia), a homeobox gene that provides a double assurance for axial and neural development in *Xenopus* (21). Nodals are members of the Transforming Growth Factor-beta (TGF-β) superfamily that function during gastrulation in most vertebrates and resemble the mesoderm inducer Activin in their biological activity (22). The rescue of β-catenin depletion by Nodals was partially mediated by the induction of *chordin*, such that

$$\begin{array}{c} \text{Hwa}\to \beta \text{catenin}\to \text{Nodals}\to \text{Chordin}. \end{array}$$

A player not addressed in previous studies was Goosecoid, a gene activated by Nodals, that was initially described as a primary response gene induced by Activin even when protein synthesis is blocked (1, 23). We had assumed that Gsc would function downstream of Nodal/Activin.

In the present study, we tested whether *gsc* mRNA could rescue the β-catenin depletion phenotype. We found that *goosecoid* mRNA was able to completely rescue the β-catenin knockdown ventralized phenotype. To our surprise, this rescue was blocked by coinjection of *lefty1* or *cerberus-Short* (*cerS*) mRNAs, which are specific secreted antagonists of Activin/Nodal and Nodals, respectively (24, 25) suggesting a possible alternative pathway:

Hwa→βcatenin→Gsc→Nodals.

In addition, in double knockdown experiments targeting both β-catenin and *gsc*, the dorsalizing activity of *Xnr6*, as well as that of *β-catenin*, displayed a strong requirement for Goosecoid. These results imply that a positive signaling loop involving Nodal → Gsc and Gsc → Nodal plays an important role in early development in this sensitized system. Together with previous experiments, the present work suggests that the early dorsal β-catenin signal activates Goosecoid, Nodals, and Siamois in parallel to resiliently induce the Spemann organizer biochemical pathway during *Xenopus* development.

## Results

### Goosecoid Rescued Axial Development After β-Catenin Depletion and Was Blocked by Lefty.

**Fig. 1.**
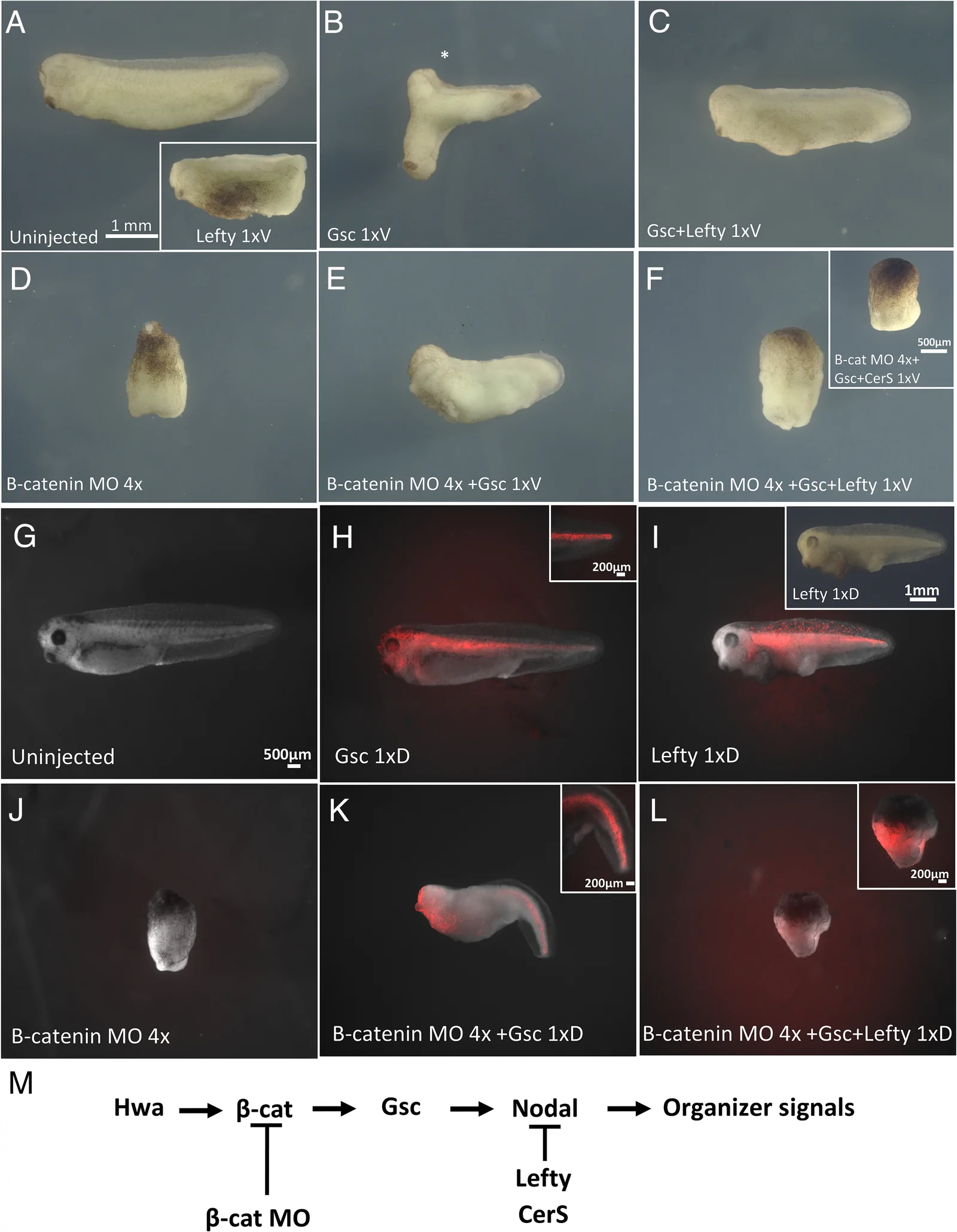
Goosecoid rescued axis formation in β-catenin-depleted ventralized embryos, and this was blocked by the Nodal/Activin inhibitor Lefty. (*A*) Uninjected control embryo (the *Inset* shows lefty-injected embryo with small head and pigmented epidermis accumulation in the belly). (*B*) A single ventral injection of mouse gsc mRNA induced an incomplete secondary axis indicated by a star. (*C*) One-time ventral (1xV) injection of lefty mRNA suppressed the secondary axis induced by mouse gsc. (*D*) β-catenin-depleted embryo exhibiting complete ventralization. (*E*) A single injection of mouse gsc mRNA into a ventral blastomere at the 4-cell stage rescued primary axis formation in embryos ventralized by β-catenin MO injection (4×). (*F*) Coinjection of lefty mRNA completely inhibited dorsal axis rescue by *gsc* mRNA resulting in ventralized β-cat embryos; the *Inset* shows that CerS mRNA also blocked axial rescue by Gsc indicating a complete requirement for Nodals. (*G*) Uninjected control embryo. (*H*) Single dorsal injection (1xD) of mouse gsc mRNA together with lineage tracer rfp mRNA showing normal dorsal organizer fates extending from anterior endomesoderm to the tip of the tail. (*I*) lefty mRNA 1xD injection caused slight ventralization. (*J*) Embryo ventralized by β-catenin depletion. (*K*) One-time dorsal injection of mouse gsc mRNA rescued a complete primary axis extending from head to tail (the *Inset* shows RFP extending to the CNH); 1xD injection resulted in higher frequency of complete rescue with cement glands and was adopted for the rest of this study. (*L*) lefty mRNA blocked axis rescue by gsc mRNA. (*M*) Proposed pathway implicating Gsc downstream of β-catenin. Numbers of embryos were as follows: (*A*) n = 68, all normal (*Inset*, n = 17, all slightly ventralized). (*B*) n = 17, 11 with incomplete second axes and six normal. (*C*) n = 43, 33 ventralized with a dark patch in the belly, 10 normal. (*D*) n = 32, 24 completely ventralized. Five weak axes with no cement gland, three with weak axis with cement gland. (*E*) n = 47, all rescued, 34 with cement gland, 13 lacking cement glands. (*F*) n = 24, 22 completely ventralized, two with weak residual axes lacking cement glands; *Inset* n = 30, all ventralized. (*G*) n = 67, all normal. (*H*) n = 36, three with enlarged cement gland, rest of normal appearance. (*I*) n = 31, 30 completely ventralized, one escaper. (*J*) n = 24, 21 completely ventralized, three weak axes lacking cement gland. (*K*) n = 113, 109 with axial rescue, four with no rescue. (*L*) n = 29, 28 completely ventralized, one escaper. Scale bar lengths are indicated. Data from five independent experiments.

Microinjection of 150 pg of mouse *gsc* mRNA (15) into a ventral blastomere induced weak incomplete secondary axes lacking head structures and cement glands in wild-type (wt) embryos (Fig. 1 *A* and *B*). However, in embryos ventralized by injecting β-catenin morpholino (β-cat MO) into each blastomere at the 4-cell stage, complete axial structures, including heads, were rescued after a single ventral (1xV) injection of *gsc* mRNA at 8-cell (Fig. 1 *D* and *E*). After 1x dorsal (1xD) injection, rescue was even more effective in terms of the frequency of cement gland with eye structures marked by *rx2a* (Fig. 1 *J* and *K* and *SI Appendix*, Fig. S1). β-catenin depleted embryos have normal cortical rotation, and the dorsal preference may indicate a currently unidentified dorsal component. For the rest of this study, we chose to use 1xD injections using mouse *gsc* mRNA, which is more active than *Xenopus* and can be used in morpholino rescue experiments (15).

Coinjection of *lefty* mRNA (500 pg) inhibited Gsc secondary axes in wt embryos after one-time ventral injection (Fig. 1 *B* and *C*). Surprisingly, in β-catenin depleted embryos, all dorsal axial structures induced by *gsc* mRNA (150 pg) were blocked by coinjection of *lefty* mRNA (Fig. 1 *D*–*F* and *J*–*L*), indicating a complete requirement for Nodal/Activin. Similarly, coinjection of *cerS* mRNA (150 pg), a specific inhibitor of Nodals (25), also blocked Gsc rescue (Fig. 1 *F*, *Inset*). As *cerS* embryos tended to have blastopore closure problems *lefty1*, which results in better epiboly, was used for the rest of this study. In *X. laevis*, there are 11 *Xenopus* nodal-related genes (*Xnrs*), many of them derived from tandem duplications of *Xnr5* and *Xnr6* (22). Lefty/Antivin is a potent secreted antagonist (member of the TGF-β family of secreted proteins) that binds to Nodal/Activin receptors but does not activate them, blocking activity of Xnr6 (*SI Appendix*, Fig. S2 *A*–*F*) and of the other Xnrs (24). Mammals have a single Nodal gene, which is not only required for gastrulation but also for left–right asymmetry. In *Xenopus*, there are multiple Nodal-related genes, which are mostly expressed only during a brief window at late blastula/early gastrula likely representing gene duplications required for the rapid activation of mesoderm induction (*SI Appendix*, Fig. S2 *G*–*O*).

In lineage tracing studies using coinjection of red fluorescent protein (*rfp* mRNA), dorsal injection of *gsc* mRNA marked all organizer derivatives such as head endomesoderm and axial derivatives, extending all the way to the CNH in the tail tip, both in wt and rescued embryos (Fig. 1 *H* and *K*). Unlike ventral injection of *gsc* mRNA, 1xD injection did not induce axial structures since it is normally expressed in the dorsal organizer (Fig. 1 *B* and *H*). *lefty* mRNA on its own reduced head structures but did not affect the tail organizer (Fig. 1*I*). Although *gsc* and *Brachyury* repress each other at neurula stages (26, 27), Gsc overexpression did not appear to affect differentiation of the late tail organizer (Fig. 1 *K*, *Inset*).

We conclude that *gsc* mRNA efficiently rescued the ventralization caused by β-catenin depletion and that in order to activate organizer signals Goosecoid requires the activity of Nodals (Fig. 1*M*).

### Endogenous Gsc Is Required for Dorsalization by Xnr6.

**Fig. 2.**
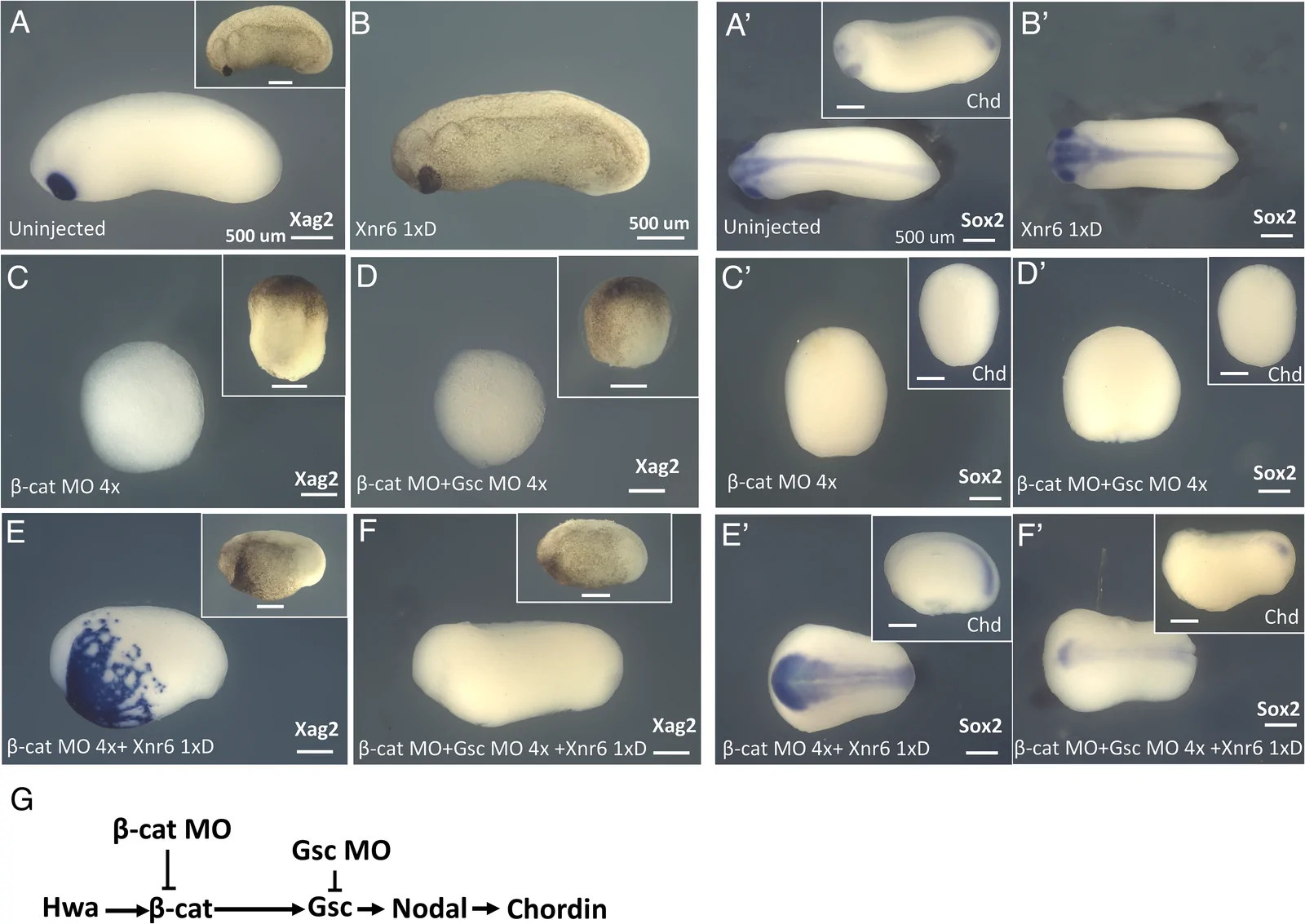
Goosecoid function was required for the complete axial rescue of β-Catenin depletion by Xnr6. (*A* and *A’*) Uninjected embryo hybridized with the cement gland (CG) marker *Xag2* or the central nervous system (CNS) marker *Sox2*. (*B* and *B’*) Single injection of *Xnr6* mRNA into a dorsal blastomere at the 4-cell stage induced a slightly enlarged CG and anterior CNS. (*C* and *C’*) Depletion of β-catenin with MO resulted in complete ventralization of embryos, lacking CG and CNS tissues. (*D* and *D’*) Embryos double depleted of β-catenin and Goosecoid with MOs were completely ventralized. (*E* and *E’*) *Xnr6* mRNA 1xD rescued and overdorsalized the primary axis in β-catenin depleted embryos marked by *Xag2*, *Sox2* and *chd*; note ectopic cement glands. (*F* and *F’*) Depletion of Goosecoid strongly suppressed dorsalization induced by *Xnr6* in β-catenin-depleted embryos. (*G*) Experimental design and proposed Spemann organizer pathway. Numbers of embryos were as follows: (*A*) n = 98, all normal. (*B*) n = 41, weakly dorsalized or normal. (*C*) n = 59, 57 completely ventralized. (*D*) n = 82, all completely ventralized. (*E*) n = 88, all with axial rescue. (*F*) n = 89, 75 with rescued axes but much weaker compared to β-catenin alone. Scale bars are indicated. Data from three independent experiments.

We next tested whether *gsc* is required for the activity of microinjected *Xnr6* mRNA. *Nodal* mRNAs injected ventrally into the wt embryo induce only weak incomplete secondary axes (19), and when injected dorsally have almost no phenotypic effect (Fig. 2 *A*–*B’*). A specific antisense morpholino that targets both alleles of *gsc* (Gsc MO) has been characterized in previous work from our laboratory (15). When coinjected 4× at 4-cell together with β-cat MO embryos, Gsc-depleted embryos were completely ventralized without any axial structures (Fig. 2 *D* and *D’*). The β-cat MO background provides a sensitized system in which a single dorsal injection of 1 pg of *Xnr6* mRNA induced robust axial formation, resulting in dorsalized embryos with very large heads including ectopic cement glands marked by *xag2* (Fig. 2*E*) (28), and enlarged brains marked by the panneural marker *sox2* (Fig. 2*E’*). When the same experiment was performed together with *goosecoid* depletion, a striking decrease of cement gland, forebrain, and eyes was observed (Fig. 2 *F* and *F’*, compared to Fig. 2 *E* and *E’* and *SI Appendix*, Fig. S3). At tailbud stages, *chd* expression in posterior notochord and CNH was decreased by Gsc MO although to a lesser extent than anterior structures (Fig. 2, *Insets* in *A*’, *E*’, and *F*’). Conversely, in overexpression experiments RT-qPCR analyses showed that *Xnr6* transcription was increased by *gsc* mRNA coinjection in β-catenin depleted embryos at mid-gastrula (*SI Appendix*, Fig. S4).

We conclude from these experiments that the dorsalizing effects of *nodal* mRNA in the embryo have a strong requirement for Gsc in the β-cat MO sensitized background and that Gsc can induce the expression of *Xnr6*.

### A Positive Feedback Loop Between Gsc and Nodals Downstream of β-Catenin.

**Fig. 3.**
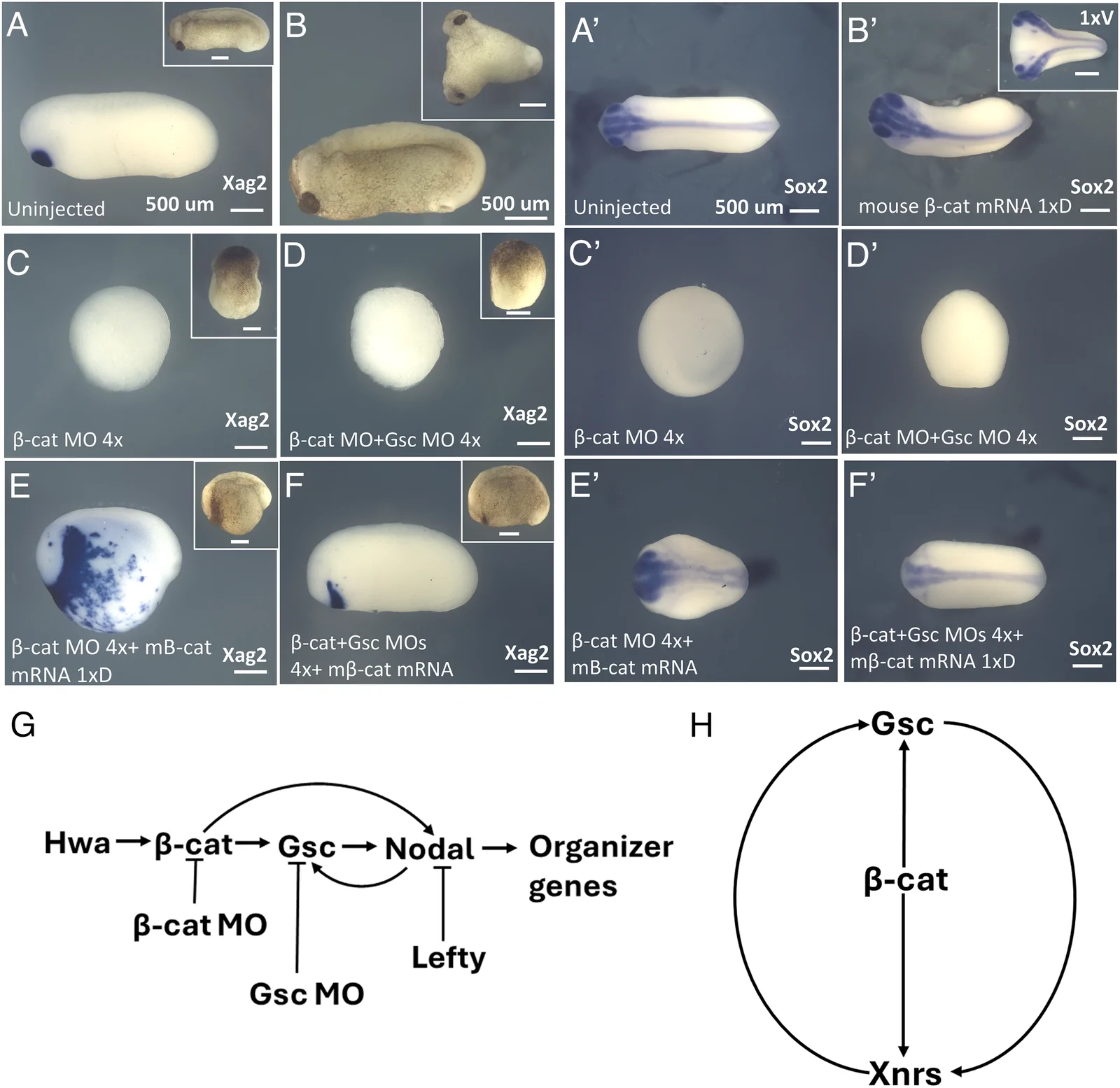
The rescue of ventralized *Xenopus* embryos by mouse *β-catenin* mRNA was attenuated by *goosecoid* depletion. (*A* and *A’*) Uninjected embryo with CG marked by *Xag2* or CNS with *Sox2*; the *Inset* shows brightfield image. (*B* and *B’*) 1xD microinjection of *β-catenin* mRNA into a dorsal side caused moderately dorsalized phenotypes; *Insets* show that single injection into ventral blastomere caused twinned complete axes. (*C* and *C’*) β-catenin depletion (4× β-catMO) resulted in complete ventralization of embryos. (*D* and *D’*) Double depletion of β-catenin and Goosecoid resulted in ventralization lacking CG and CNS. (*E* and *E’*) *β-catenin* mRNA rescued axis induction resulting in ectopic CGs and strong dorsalized phenotypes with enlarged brains in β-cat MO embryos. (*F* and *F’*) Goosecoid is required for the robust dorsalization driven by *β-catenin* mRNA in β-catenin-depleted embryos. (*G*) Pathway of dorsal development in *Xenopus* suggested by this figure. (*H*) The main conclusion of this paper: A positive feedback loop between Goosecoid and Nodals exists in Spemann–Mangold organizer tissue. Number of embryos analyzed: (*A*) n = 78, all normal. (*B*) n = 51, seven dorsalized others mostly normal; *Inset*: n = 61, 30 complete second axis, 25 incomplete second axes. (*C*) n = 52, all completely ventralized. (*D*) n = 37, all completely ventralized. (*E*) n = 74, 64 strongly dorsalized with large CGs (12 with ectopic cement glands), seven rescued without CG, three ventralized escapers. (*F*) n = 52, 36 almost normal axes with CGs but not dorsalized, three with enlarged but no ectopic CGs, nine partially rescued but lacking CG, three completely ventralized. Scale bar length 500 µm; three independent experiments.

Next, we tested whether the dorsalizing effect of β-catenin had a requirement for Gsc function. To analyze this, we microinjected a stabilized form of murine *β*-*catenin* (m*β*-*catenin*) mRNA that is not targeted by the β-cat MO (29). In wt embryos 1xD microinjection of 150 pg m*β*-*catenin* mRNA resulted in only modest expansion of anterior brain structures marked by *sox2* while 1xV injection induced secondary axes, including many complete ones (Fig. 3 *A*–*B’*). In β-catenin depleted embryos, the same amount of *mβ-catenin* mRNA microinjected 1× dorsal at 8-cell induced strongly dorsalized phenotypes with ectopic cement glands and enlarged brain structures marked by *xag2* and *sox2*, while embryos also depleted of Goosecoid were rescued only to the level of wt embryos or less (Fig. 3 *C*–*F’*).

A developmental pathway in which endogenous β-catenin induces both Gsc and Nodal in parallel is proposed in Fig. 3*G*. The main conclusion from these experiments is that a positive feedback loop between Gsc and Nodals regulates Spemann organizer formation, particularly in anterior regions of the embryo (Fig. 3*H*).

## Discussion

**Fig. 4.**
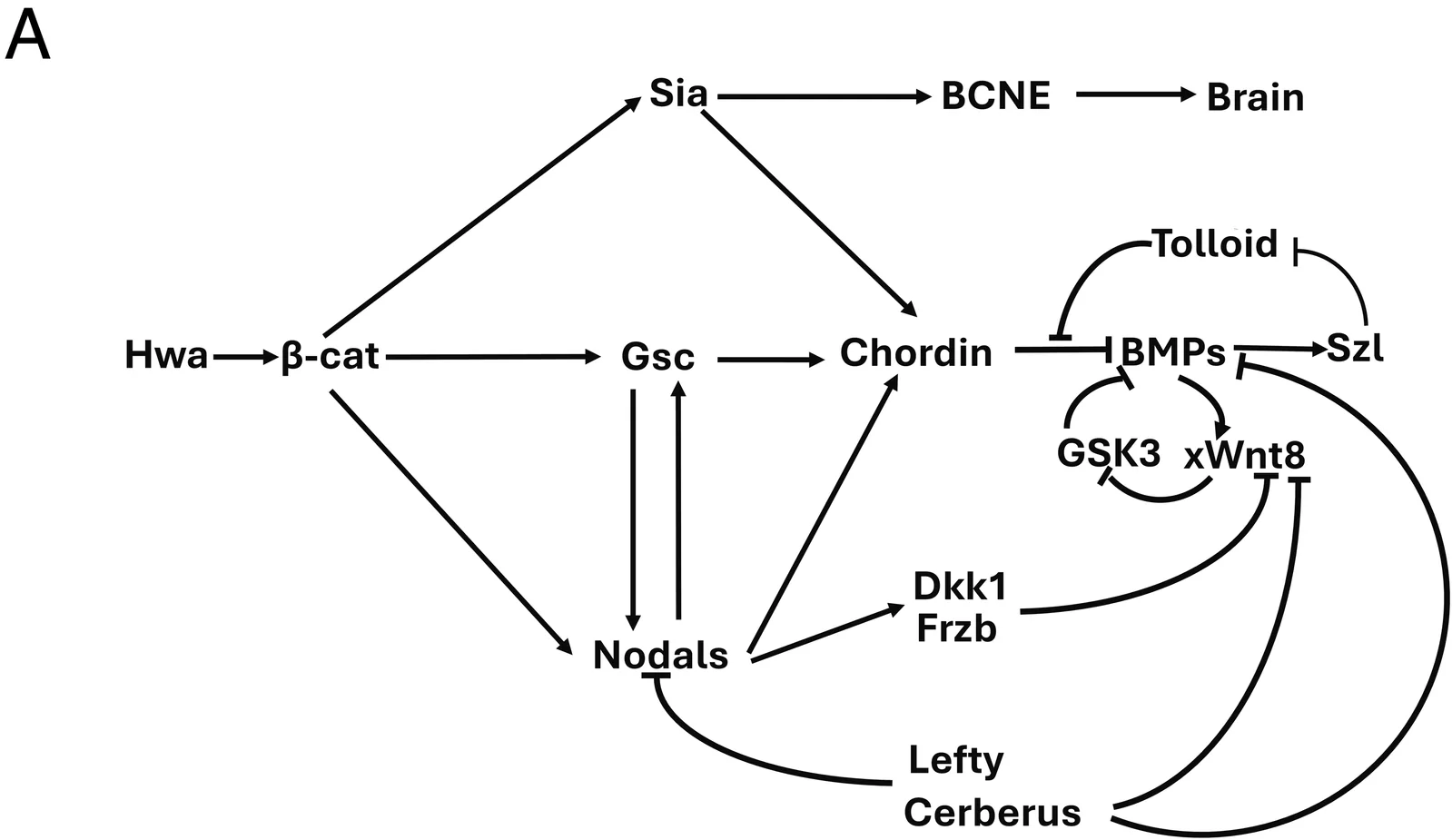
Proposed signaling pathway for *Xenopus* axial development deduced from the present epistatic study and two previous ones (19, 21). (*A*) The maternal cytoplasmic determinant Huluwa (Hwa) protein stabilizes β-catenin-dependent transcription on the dorsal side. Dorsal β-catenin directly initiates two parallel signaling centers through the activation of Siamois and Nodal genes to induce the Blastula-Chordin-Noggin-Expression (BCNE) and Nieuwkoop centers, respectively. The present study uncovered a reciprocal positive feedback loop between Nodals and Goosecoid. Nodals are increased by Goosecoid in addition to β-catenin, and Nodals induce Goosecoid. This positive regulatory circuit ensures robust axis specification in *Xenopus*. Both the Nodal- and Siamois-dependent pathways converge to transcriptionally activate a largely overlapping set of Spemann–Mangold organizer genes that include multiple secreted factors in addition to the BMP antagonist Chordin shown here (21). The direct transcriptional activation of chordin by Goosecoid has been demonstrated recently (33). In the ventral side, BMP signaling restricts organizer activity. BMP signaling induces zygotic xWnt8, which further amplifies BMP signaling by inhibiting GSK3 and increasing Smad1/5/8 signaling (34), forming a ventral positive feedback loop that is inhibited by the organizer antagonists Dkk1 and Frzb. BMP also induces Sizzled (Szl), a secreted inhibitor of the Tolloid protease responsible for Chordin degradation, establishing a negative feedback mechanism that stabilizes Chordin levels.

In this study, we asked whether Gsc played a role in the epistatic signaling pathway of dorsal development. To our surprise, *gsc* mRNA was able to completely rescue the ventralization caused by β-catenin depletion. As reported previously, and indicated in Fig. 4, complete dorsal axes can be rescued in β-cat MO embryos by single microinjections of *sia*, *nodals*, or *chd* mRNAs (19, 21). We expected Gsc to be downstream of Nodals, for these are the growth factors that mediate Gsc induction in mesoderm by Nieuwkoop center signals secreted by dorsal endoderm in the *Xenopus* blastula (30). We did not expect Gsc would rescue a complete Spemann organizer. Rescue by *gsc* mRNA was blocked by coinjection of *lefty* mRNA, a secreted inhibitor of Nodal/Activin receptors (24) and by CerS, a secreted antagonist that binds nodals in the extracellular space (25). In zebrafish, overexpression of the *nodal-related* genes *cyclops* and *squint* leads to *goosecoid* induction and, conversely, MO knockdown of these genes blocks *chd* expression (31). In addition, attenuation of Nodal signaling by increasing concentrations of the Nodal/Activin antagonist Lefty/Antivin results in the inhibition of *goosecoid* expression (32).

In *Xenopus* and mouse, the *gsc* promoter contains a distal promoter element inducible by Nodal/Activin, and a proximal element activated by β-catenin (35). In the context of the wild-type *Xenopus* embryo *gsc* mRNA had only weak axis induction activity but caused strong dorsal induction in β-catenin depleted embryos (Fig. 1). Similarly, the dorsalizing activity of *Xnr6* was weak in wt embryos, but very potent in the sensitized β-cat MO background. Experiments using Gsc MO revealed a strong requirement for both *Xnr6* (Fig. 2 *E*–*F’*) and mouse *β-catenin* mRNA dorsalizing activities in the β-catenin sensitized system (Fig. 3 *E*–*F’*). As this study shows, a positive feedback loop between Gsc and Nodals exists downstream of β-catenin (Fig. 4).

Positive feedback is a fundamental biochemical mechanism to attain novel cell states, from bacteriophage lambda lysogeny to induced pluripotent stem cells (36). Gsc promotes anterior cell migration in *Xenopus* endomesoderm (3), is required for craniofacial development after gastrulation (11), and promotes epithelial–mesenchymal transition (EMT) in cancer metastases (37, 38). The Gsc/Nodal regulatory loop reported here functions in the establishment of an early Spemann organizer state (*SI Appendix*, Fig. S2 *G*–*N*). At the molecular level, this functional interaction is reflected in the ability of Gsc to induce downstream organizer genes such as Chordin and other organizer genes. Gsc is generally a transcriptional repressor (5, 39) and is able to directly bind to the *chd* promoter (33, 40). Gsc can also function as a transcriptional activator when monoubiquitinylated (41), and in the case of the *chordin* promoter increases transcription (5). As shown in *SI Appendix*, Fig. S4, Gsc upregulates *Xnr6* transcription at gastrula. However, these results do not distinguish whether Gsc is a transcriptional activator or an inhibitor of ventral signals such as Vent1/2 (15). The *chd* promoter also contains multiple overlapping Smad4 binding sites that respond to Activin/Nodal signaling (42).

In Fig. 4 only Chordin is indicated downstream of Sia, Gsc, and Nodals, but many other secreted factors are also expressed in Spemann organizer and collectively contribute to dorsal patterning (43). In agreement with this functional redundancy, knockdown of *chd* using antisense morpholino oligonucleotides (Chd MO) has strong but partial effects on dorsal development, indicating that Chd acts as an important but not exclusive regulator of the organizer pathway (19, 21). In zebrafish, *gsc* mRNA microinjection induces complete secondary axes, and this activity is only slightly reduced in *chordino* mutant embryos and Gsc secondary axes are induced even when Noggin1 and Follistatin-like 1b were inhibited with MOs (44). While the positive feedback loop between Gsc and Nodals could explain in part the mild loss-of-function phenotype of Gsc, parallel pathways, including other homeobox genes, probably also cooperate (45, 46). Definitive evidence would require double and triple MO knockdowns for *gsc*, *sia*, *Xnr5,* and *Xnr6*, which are difficult because these genes are present in multiple copies in the *Xenopus* genome (21).

The function of Chordin is to antagonize ventral differentiation driven by BMP4 signaling (Fig. 4). The rate-limiting step in the D-V pathway is the proteolytic cleavage of Chordin by the Tolloid metalloproteinase resulting in the shuttling of active BMPs (47, 48). In the ventral side, Sizzled, a BMP4-induced secreted factor induced at high levels of BMP4, functions as a competitive inhibitor of Tolloid, forming a negative feedback loop that stabilizes BMP4/pSmad1/5/8 signaling levels (19, 49). BMP4 also induces xWnt8 expression, which enhances Smad1/5/8 activity by inhibiting GSK3 in ventrolateral regions (34). Dorsal signals mediated by the secreted Wnt antagonists Dickkopf1 (Dkk1) and Frzb limit ventral xWnt8 signals (19). This self-organizing molecular signaling network provides a robust dorsoventral patterning system during early embryogenesis.

While the regulatory interactions in Fig. 4 are supported by *Xenopus* epistatic studies, organizer-associated transcriptional programs have also undergone evolutionary specialization. In zebrafish, the *bozozok*/*dharma* homeobox gene plays a crucial role during gastrulation and is expressed in the yolk cell and deep endoderm (50, 51). Bozozok opposes the activity of the ventral *vent* (*vega*) homeobox genes in zebrafish (52). Interestingly, the *bozozok/dahrma* transcriptional repressor evolved from a Goosecoid homeodomain protein ancestor that became independent of Nodal signaling but maintained activation by dorsal β-catenin (21). In *bozozok* mutants, expression of zebrafish *nodals* is greatly reduced, resulting in head defects (53). In *Xenopus*, another homeobox gene, *siamois*, is a transcriptional activator induced solely by β-catenin and independent of Nodal/Activin signaling. Siamois evolved from *sebox* (skin-embryonic-brain-oocyte-specific-homeobox), a different homeodomain protein branch derived from the Mix1 family of Activin primary response genes (54, 55). The *siamois* gene is initially expressed in the dorsal animal cap region where it directs the formation of the Blastula-Chordin-Noggin-expression (BCNE) center (4, 56). Sia, which exists in seven copies in *X. laevis*, may represent an evolutionary adaptation for rapid brain induction in frogs. It is interesting that both Siamois and Bozozok lost the ability to be induced by Nodal/Activin possessed by their ancestors (Mix1 and Gsc) but retained the entire spectrum of downstream organizer target genes (21).

In the chick embryo, *gsc* expression is initially detected in Koller’s sickle at the posterior of the unincubated egg (57), while *chd* expression is detectable in a central population of epiblast cells just anterior to the sickle that later contributes to brain formation like *Xenopus* Sia (58, 59). This central population might be functionally analogous to the *Xenopus* Siamois-induced Blastula Chordin and Noggin Expression (BCNE) center in prospective brain ectoderm, but no specific chick homeobox gene has yet been associated with it (4). The mouse organizer, despite normal embryonic gastrulation, has impaired neural inductive activity when *goosecoid*^−/−^ mouse nodes (anterior primitive streak at day 7) are transplanted into early chick embryos; even gsc^+/−^ nodes showed decreased *Sox2* induction (60). Taken together, these findings highlight the evolutionary flexibility and functional conservation of homeobox genes in gastrulation. While different species have recruited the expression of homeobox genes like *bozozok* and *sia* for species-specific patterning, the role of Goosecoid as a core organizer gene has remained remarkably conserved from *Hydra* through annelids to vertebrates (7, 61).

In summary, recent epistatic studies in *Xenopus* have defined a signaling pathway governing dorsal axis formation (19, 21) (Fig. 4). The present study placed Goosecoid, the archetypal organizer marker, within this pathway and revealed a positive feedback loop between Nodal and Goosecoid acting downstream of Hwa and β-catenin. Functional redundancy within this network may help explain the mild phenotypes observed by *gsc* loss-of-function despite its remarkable evolutionary conservation. Developmental robustness requires resilient, self-organizing signaling circuits to ensure reproducible axial embryonic patterning.

## Materials and Methods

### *Xenopus* Ethic Statements.

All experiments involving *Xenopus* were performed in compliance with the NIH Guide for the Care and Use of Laboratory Animals. Experimental protocols were reviewed and approved by the Institutional Animal Care and Use Committee (IACUC) of the University of California, Los Angeles, Medical School (Permit No. ARC-1995-129). Adult pigmented female and albino male *X. laevis* specimens were obtained from *Xenopus*-1 Inc. (Dexter, MI).

### mRNA Synthesis.

pCS2-HA-RFP, pCS2-antivin/lefty1, and pCS2-Xnr6 plasmids were linearized with NotI, whereas pCS2-mouseGsc was digested using XhoI. Capped mRNAs were synthesized using the mMessage mMachine SP6 kit containing SP6 RNA polymerase (Invitrogen, MA), followed by purification with the MegaClear kit (Invitrogen, MA). The synthesized mRNAs were injected either ventrally or dorsally into a single blastomere at the 4- or 8-cell stage in a final volume of 4 nL. Microinjected mRNAs were titrated in embryos and used in the following amounts: *Xnr6* mRNA 1 pg, *gsc* mRNA 150 pg (15), *lefty* mRNA 250 pg (62), mouse *β-catenin* 150 pg (29), and HA-RFP 100 pg (29).

### Morpholinos.

Antisense morpholino oligonucleotides against *X. laevis* β-*catenin* (5′-TTTCAACCGTTTCCAAAGAACCAGG-3′) and *gsc* (5′-GCTGAACATGCCAGAAGGCATCACC-3′) were obtained from Gene Tools, LLC (Philomath, OR) (13, 20). For knockdown experiments, 10 ng of β-cat MO or 20 ng of Gsc MO were injected into each blastomere at 4-cell (resulting in total amounts of 40 ng and 80 ng per embryo, respectively) at the 2- to 4-cell stage.

### *Xenopus* Embryo Manipulations and Whole Mount In Situ Hybridization.

Eggs were fertilized as in vitro using freshly dissected testis in 1× MMR (Marc’s Modified Ringer’s solution: 58.44 g/L NaCl, 1.49 g/L KCl, 2.94 g/L CaCl_2_/2H_2_O, 0.95 g/L MgCl_2_, 11.94 g/L HEPES, pH = 7.4 adjusted using NaOH). Following fertilization, eggs were incubated for 5 min at room temperature. Subsequently, 0.1× MMR was added, and the dishes were maintained at RT for an additional 70 min. To remove the jelly coat, fertilized embryos were treated with 2% cysteine in 0.1× MMR (pH 8.0) for 10 min at RT and rinsed with 0.1× MMR three times. Embryos were then microinjected in 1× MMR on 2% agar-H_2_O coated Petri dishes. After injection, embryos were transferred to agar-coated dishes containing 0.1× MMR and were incubated at room temperature until fixation. Embryos were fixed overnight at 4°C in 4% paraformaldehyde (PFA) in phosphate-buffered saline (1x PBS diluted from 10x PBS: Biotech, PD8117) in 1 dram glass vials (Fisherbrand, FS60940d-1). Whole-mount in situ hybridizations used a modification of the original Richard Harland protocol. Following fixation, embryos were dehydrated through a methanol series and stored in 100% methanol at -20°C overnight. Embryos were bleached in 2:1 MeOH:H2O2 (30% w/w Sigma) overnight under bright fluorescent light above aluminum foil. For rehydration, embryos were sequentially incubated in 75%, 50%, and 25% methanol series in PBS for 5 min each at room temperature (RT). Embryos were then washed three times for 5 min each in PBS containing 0.1% Tween-20 (PBSw) at room temperature (RT). Subsequently, embryos were incubated for 5 min at RT in a 1:1 mixture of hybridization buffer (10g Blocking reagent from Roche/Sigma cat# 11 096 176 001, 500mL Formamide, 250mL 20x SSC (175.3g NaCl, 77.4g Sodium citrate in autoclaved dH2O pH:7.0), 120mL autoclaved dH2O, 100mL 1g/100mL torula RNA from Roche/Sigma cat# 10109509001, 2mL Heparin (50mg/mL in 1xSSC), 5mL 20% Tween20, 10 ml 10% CHAPS detergent,10mL 0.5M EDTA) and PBSw, followed by incubation in 100% hybridization buffer for 3 h at 70°C. Embryos were then incubated overnight in a water bath at 70°C with 1 mL antisense RNA probes diluted to 1 ng/µL in hybridization buffer. The following day, the probe solution was removed and stored, and embryos were washed in hybridization buffer, followed by three washes in 2x SSC buffer for 5 min each at 70°C. Embryos were then washed twice for 30 min each at 70°C in 2x SSC containing 0.1% CHAPS, followed by two washes in 1x MAB (maleic acid buffer diluted from 5x solution: 58g Maleic acid, 43.65g NaCl, pH to 7.5 with NaOH, dH2O up to 1L) for 30 min each at 70°C. Embryos were subsequently washed twice in PBSw for 10 min at RT. Following washing, embryos were incubated in blocking solution (Roche cat# 11096176001 in 1x MAB) for 1 h at 4°C with gentle shaking. The antibody solution was replaced with fresh antibody solution prepared in blocking buffer as 1:10,000 dilution, and embryos were incubated overnight at 4°C with gentle shaking. Following antibody incubation, embryos were washed five times for 1 h each at RT in PBSw containing 0.1% bovine serum albumin (BSA). Embryos were then washed twice in PBSw for 30 min each at RT. Subsequently, embryos were incubated twice in AP1 buffer (0.1M NaCl, 0.1M Tris-HCl pH 9.5, 50mM MgCI2) for 10 min each at RT. For color development, embryos were incubated overnight at 4°C in 1 mL BM purple substrate (Roche/Sigma-Aldrich, Cat. No. 11442074001). The staining reaction was terminated by washing embryos with stop solution (100mM Tris-HCI pH 7.4 5mL, 1mM EDTA), after which the embryos were again dehydrated stepwise in methanol, stored at -20°C, rehydrated, and imaged. Imaging was conducted using a ZEISS Axio Zoom.V16 stereomicroscope equipped with Z-stack functionality to obtain three-dimensional image data.

### qRT-PCR.

Quantitative real-time RT-PCR (RT-qPCR) was conducted at stages 10 and 11.5 using a previously established protocol (63). The primer sequences utilized for the qRT-PCR analysis were

**Table:** 

Gene	Forward primer	Reverse primer
*odc*	CAGCTAGCTGTGGTGTGG	CAACATGGAAACTCAC ACC
*chordin*	CCTCCAATCCAAGACTCC AGCAG	GGAGGAGGAGGAGCTT TGGGACAAG
*xnr6*	AGATTGAGGAAAGTACC AGTTT	GCTTTCCATTATCCTTT GGC

## Supplementary Material

Appendix 01 (PDF)

## Data Availability

All data in this study are included in the article and/or *SI Appendix*. No new software was generated in the present study.
