# Supplementary material for: Downstream of β-catenin: A positive feedback loop between Goosecoid and Nodals in Spemann’s organizer
Source: Proc Natl Acad Sci U S A. 2026 Sep 24;123(39):e2610127123. doi: 10.1073/pnas.2610127123 (PMC13624635; doi:10.1073/pnas.2610127123)
Supplement: Supplementary file 1 [file pnas.2610127123.sapp.pdf]

Supplementary Information for

Downstream of β-Catenin: a Positive Feedback Loop Between Goosecoid and Nodals in Spemann's Organizer.

Yagmur Azbazdar[a], and Edward M. De Robertis[a1]

To whom correspondence may be addressed. Email: ederobertis@mednet.ucla.edu

This PDF file includes:

Supplementary Information Figs. S1 to S4

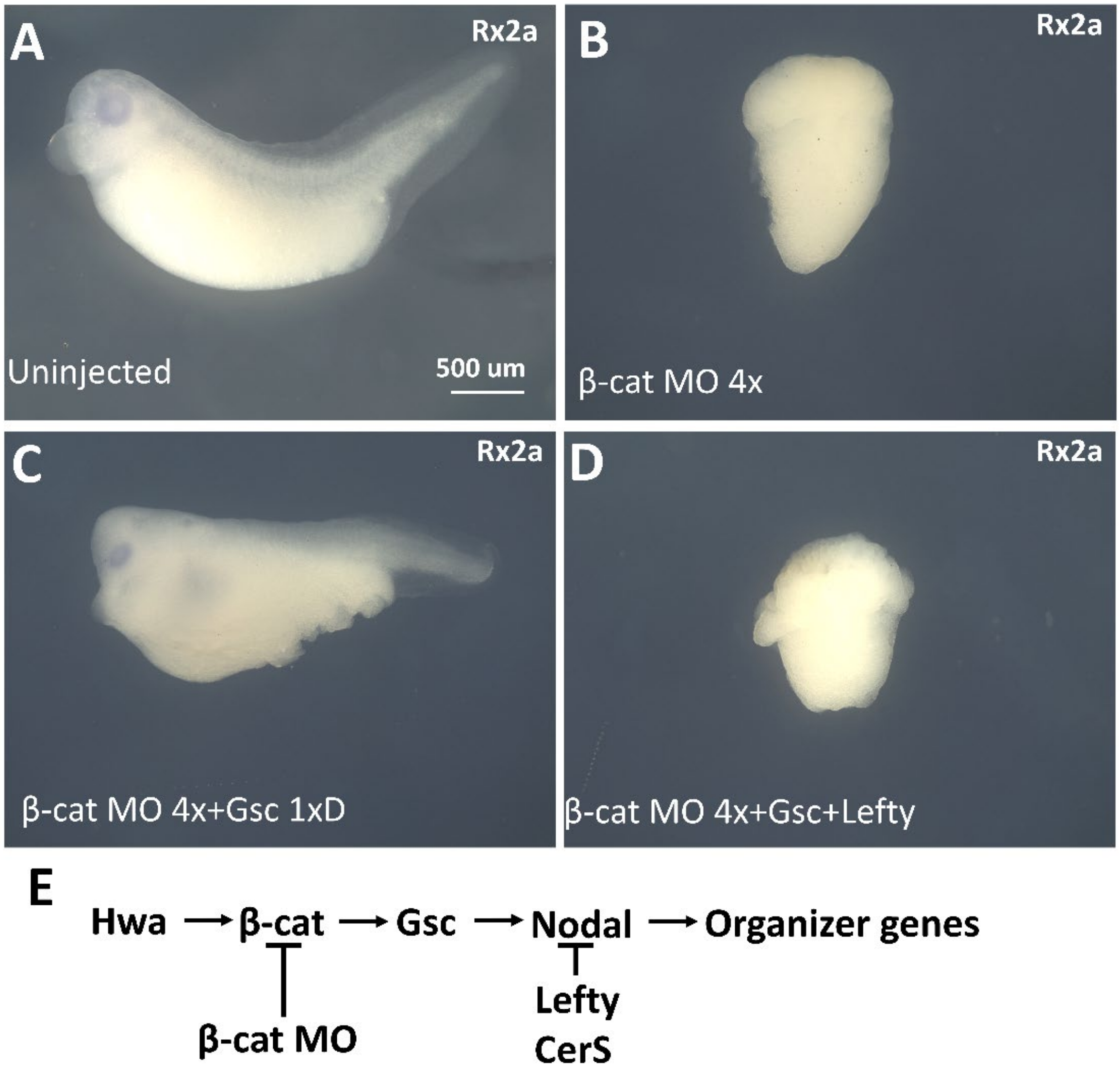

SI Appendix, Fig. S1. Goosecoid rescued head structures including eyes in β-catenin-depleted ventralized embryos and this can be blocked by the Nodal/Activin inhibitor Lefty. In situ hybridizations using the eye marker *rx2a*. (*A*) Uninjected embryos. (*B*) β-catenin depleted embryo with complete ventralization. (*C*) *mGsc* mRNA injection one time dorsally rescued complete axes marked by *rx2a*. (*D*) The Nodal/Activin antagonist Lefty blocked axial rescue by Goosecoid. (*E*) Pathway suggested by this experiment. Number of injected embryos: (*A*) n=72, all normal. (*B*) n=66, 55 completely ventralized, 8 had axis no cement gland (CG) and 3 had axis with tiny CG. (*C*) n=121, 61 rescued with CG, 47 rescued but no CG, 3 with tiny tail axes, 10 no rescue. (*D*) n=53, 51 completely ventralized and 2 had small axes. When injected 1x ventrally, *gsc* mRNA rescued axes in β-catenin depleted embryos but none had CGs in this experiment, n=15.

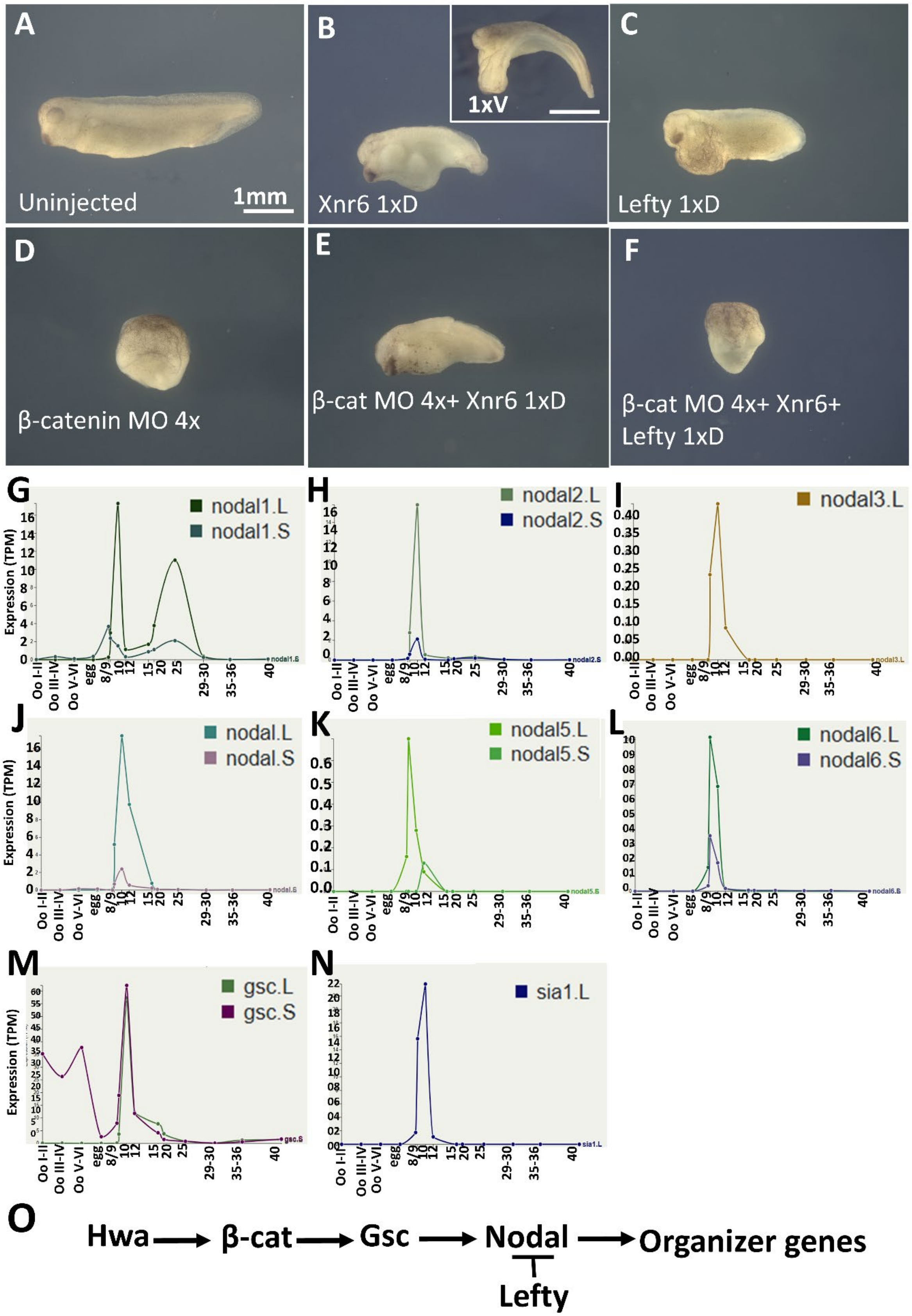
A
Uninjected
1mm
B
1xV
Xnr6 1xD
C
Lefty 1xD
D
β-catenin MO 4x
E
β-cat MO 4x+ Xnr6 1xD
F
β-cat MO 4x+ Xnr6+ Lefty 1xD
G
nodal1.L
nodal1.S
Expression (TPM)
H
nodal2.L
nodal2.S
I
nodal3.L
J
nodal.L
nodal.S
Expression (TPM)
K
nodal5.L
nodal5.S
L
nodal6.L
nodal6.S
M
gsc.L
gsc.S
Expression (TPM)
N
sia1.L
Oo I-II
Oo III-IV
Oo V-VI
egg
8/9
10
12
15
20
25
29-30
35-36
40
O
Hwa
β-cat
Gsc
Nodal
Organizer genes
Lefty

SI Appendix, Fig. S2. The antagonist Lefty blocked the activity of Xnr6 in rescuing β-catenin-depleted embryos; many β-catenin target genes are expressed in a narrow period of development at late blastula and early gastrula in *Xenopus laevis*. (*A*) Uninjected sibling embryo. (*B*) *Xnr6* mRNA 1x dorsal injection caused dorsalization; inset shows that 1x ventrally *Xnr6* induces incomplete secondary axes lacking heads in wt embryos. (*C*) Ventral injection of *lefty* mRNA reduced head structures shortened axes, and increased ventral pigmented epidermis. *(D)* 4x β-catenin MO caused complete ventralization. (*E*) A single dorsal injection of *Xnr6* mRNA at 4-cell restored complete primary axis formation in β-catenin-depleted embryos. (*F*) Co-injection of *lefty* mRNA fully prevents rescue mediated by *Xnr6 mRNA*. (*G-N*) Publicly available RNA-seq data from the Xenbase database showing that Nodals, Gsc and Sia are expressed in a narrow window of a development just before and during early gastrulation. (*O*) Pathway being analyzed. Embryo numbers analyzed were (*A*) n=40, all normal. (*B*) n=27, 24 bigger heads 3 with two cement glands. Inset: n=17, 13 with small incomplete second axis, 1 double complete axis, three no axis. (*C*) n= 40, they are ventralized with dark epidermis in the belly. (*D*) n=34, 5 complete ventralization, 20 with doi=1 no cement gland, 9 with doi=2 with cement gland. (*E*) n=31. All rescued with a large cement gland and big head. (*F*) n=35, all completely ventralized; one experiment.

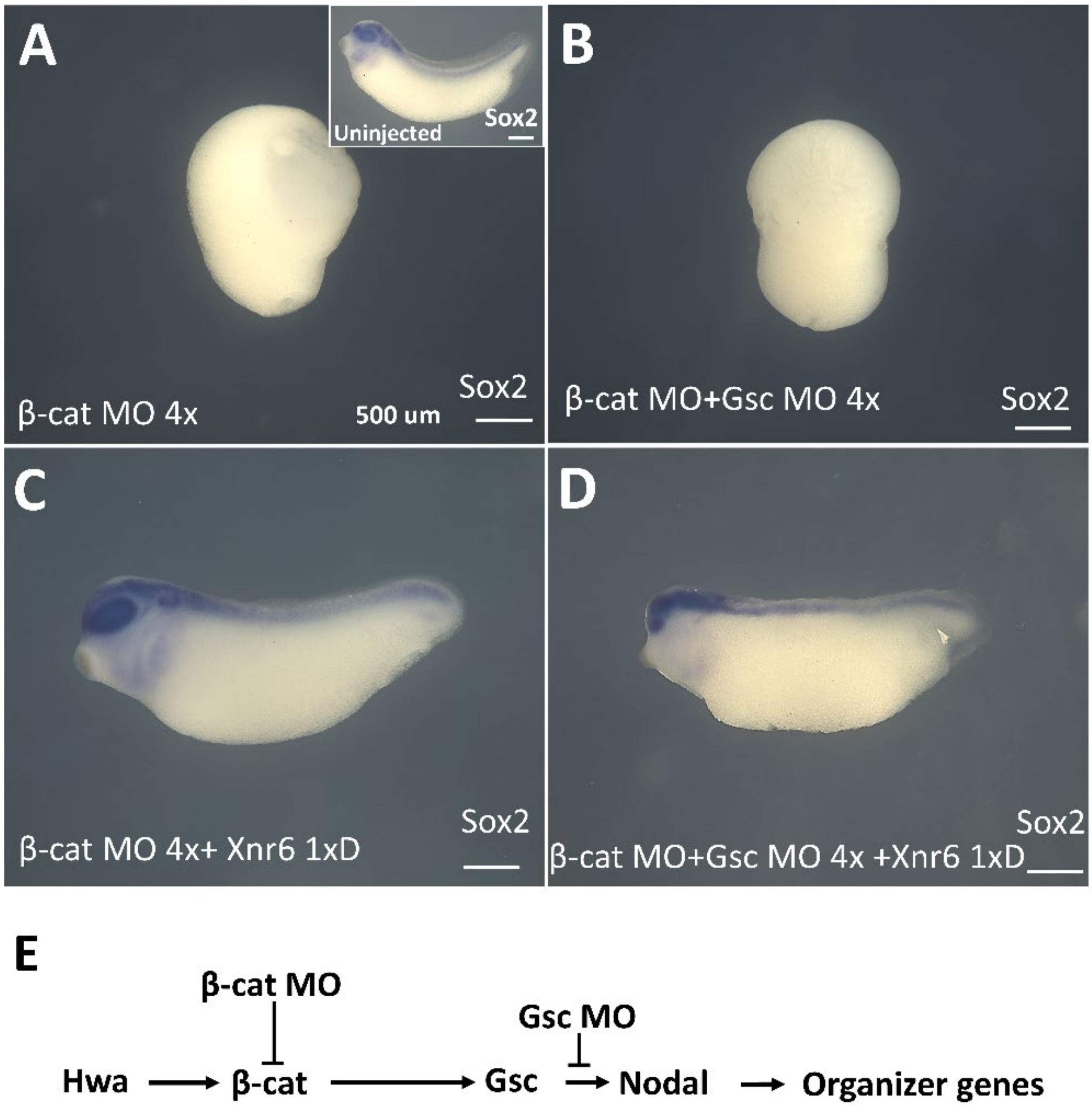

SI Appendix, Fig. S3. Goosecoid depletion inhibited the effect of exogenous *Xnr6* mRNA on anterior rescue activity in β-catenin depleted embryos. In situ hybridizations with *sox2* pan-neural markers. (*A*) β-catenin depleted embryos lacking CNS; inset shows a wt sibling. Control embryo. (*B*) Double depletion of Goosecoid and β-catenin results in ventralized embryos lacking axes. (*C*) Xnr6 completely rescued CNS formation including eyes. *Xenopus gsc* injection caused more normal embryo phenotypes. (*D*) Goosecoid is partially required for the brain-rescue activity of Xnr6. For embryo numbers see Fig. 2.

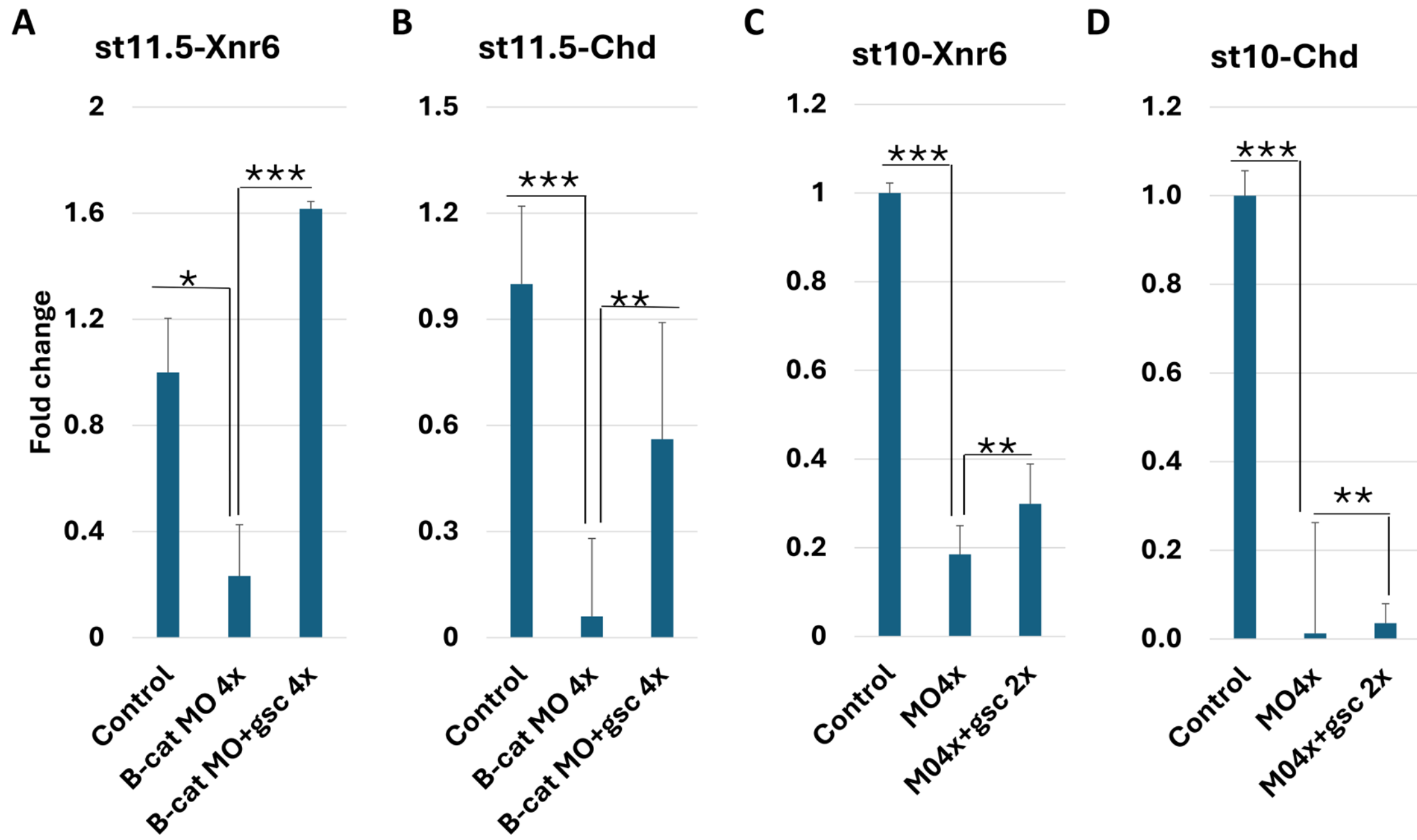

SI Appendix, Fig. S4. qRT-PCR analyses showing that *gsc* mRNA induced *Xnr6* and *chordin* transcripts at mid-gastula (stage 11.5). (*A*, *B*) Coinjection of *gsc* mRNA (150 pg) was able to induce *Xnr6* and the organizer gene *Chordin* in β-catenin MO-injected embryos. (*C*, *D*) Increased *Xnr6* and *chordin* expression started to be detectable at stage 10. Error bars indicate standard deviation (SD) (****p*<0.001, ***p*<0.01, **p*<0.05).
